# Quasi-Optimal Path Convergence-Aided Automorphism Ensemble Decoding of Reed–Muller Codes

**DOI:** 10.3390/e27040424

**Published:** 2025-04-14

**Authors:** Kairui Tian, He Sun, Yukai Liu, Rongke Liu

**Affiliations:** 1 School of Electronic and Information Engineering, Beihang University, Beijing 100191, China; philtian@buaa.edu.cn (K.T.); sunele@nus.edu.sg (H.S.); ykliu@buaa.edu.cn (Y.L.); 2 Department of Electrical and Computer Engineering, National University of Singapore, Singapore 119077, Singapore; 3 Shenzhen Institute, Beihang University, Shenzhen 518063, China

**Keywords:** Reed–Muller codes, successive cancellation decoding, automorphism ensemble decoding, early termination

## Abstract

By exploiting the rich automorphisms of Reed–Muller (RM) codes, the recently developed automorphism ensemble (AE) successive cancellation (SC) decoder achieves a near-maximum-likelihood (ML) performance for short block lengths. However, the appealing performance of AE-SC decoding arises from the diversity gain that requires a list of SC decoding attempts, which results in a high decoding complexity. To address this issue, this paper proposes a novel quasi-optimal path convergence (QOPC)-aided early termination (ET) technique for AE-SC decoding. This technique detects strong convergence between the partial path metrics (PPMs) of SC constituent decoders to reliably identify the optimal decoding path at runtime. When the QOPC-based ET criterion is satisfied during the AE-SC decoding, only the identified path is allowed to proceed for a complete codeword estimate, while the remaining paths are terminated early. The numerical results demonstrated that for medium-to-high-rate RM codes in the short-length regime, the proposed QOPC-aided ET method incurred negligible performance loss when applied to fully parallel AE-SC decoding. Meanwhile, it achieved a complexity reduction that ranged from 35.9% to 47.4% at a target block error rate (BLER) of $10^{-3}$, where it consistently outperformed a state-of-the-art path metric threshold (PMT)-aided ET method. Additionally, under a partially parallel framework of AE-SC decoding, the proposed QOPC-aided ET method achieved a greater complexity reduction that ranged from 81.3% to 86.7% at a low BLER that approached $10^{-5}$ while maintaining a near-ML decoding performance.

## 1. Introduction

Reed–Muller (RM) codes are among the most classical linear block codes, with a rich history in both academia and industry [1,2]. In recent years, RM codes have regained significant interest, potentially due to their well-qualified structures similar to those of polar codes [3]. RM codes have been proven to achieve the channel capacity over various channels, such as the binary erasure channel [4], the binary symmetric channel [5], and the binary memoryless symmetric channel [6]. Additionally, RM codes are well-known for their impressive maximum likelihood (ML) performance in the short-length regime, making them competitive for practical applications, such as the emerging hyper-reliable low-latency communication (HRLLC) [7]. Driven by these theoretical advancements and promising application potential, designing effective RM decoders has become a trending topic, leading to innovative efforts, such as minimum-weight parity check (MWPC)-aided iterative decoding [8], recursive projection-aggregation (RPA) decoding [9], and permutation-based decoding [10,11].

Most recently, a permutation-based automorphism ensemble (AE) decoder has been developed for RM codes, exploiting their rich automorphisms to achieve a near-ML performance for short code lengths [11]. The AE decoder utilizes permutations sampled from the automorphism group of RM codes [12] to produce a list of permuted channel outputs, each independently decoded by a constituent polar or RM decoder. Using a predefined metric, the AE decoder selects the most likely codeword estimate, benefiting from a diversity gain that arises from observing multiple decoding results [13]. From a practical standpoint, the successive cancellation (SC) decoder [3] is appealing as the constituent decoder due to its low complexity and efficient implementation [14,15]. For short RM codes, the AE-SC decoder demonstrates comparable performance to the SC list (SCL) decoder [16] with the same list size. Notably, the AE-SC decoder avoids the complex path management inherent in the SCL decoder [17], thereby achieving reduced overall complexity and latency [18].

However, the AE-SC decoder still faces a major complexity challenge, as its diversity gain relies on a list of SC decoding attempts. To address this issue, three early termination (ET) techniques are proposed in [10], namely, the branch and bounds (BB), repetition handling (RH), and path metric (PM) threshold (PMT)-aided methods. Additionally, the authors in [10] introduce an ET gain (ETG) metric, which is defined as the complexity ratio of the original AE-SC decoder to the AE-SC decoder incorporating an ET technique, to quantify the effectiveness of the ET method. The BB and RH methods are effective only when the SC decoding attempts are performed sequentially. The BB method exploits the monotonicity of PM updates [19] to terminate an SC decoding process if its partial PM (PPM) is already inferior to the previously best PM. Since the path with the best PM will not be terminated by the BB criterion, no performance loss is incurred compared with the original AE-SC decoder. The RH method leverages channel-dependent path diversity to skip further SC decoding attempts if the currently optimal codeword estimate, which holds the currently best PM, has been generated multiple times. By employing a heuristically selected repetition threshold, the RH method achieves a significant ETG in high signal-to-noise ratio (SNR) regions.

Despite their effectiveness, both the BB and RH methods introduce variable decoding latency and suffer from high worst-case decoding latency due to the sequential execution of all SC decoding attempts, which is undesirable in practical decoder implementations [20]. In contrast, the PMT-aided ET method supports low-latency parallel AE-SC decoding by terminating an SC decoding path when its PPM exceeds a preset threshold, which is designed to occur with a low probability for the correct SC decoding path. However, as the SNR increases, the PMT-aided ET method tends to become ineffective because most SC decoding paths can successfully decode the corrupted codeword while rarely generating PM values that exceed the threshold. Furthermore, this PMT-aided ET method focuses solely on the PM behavior of each SC decoding path independently without considering the collective features of PMs across multiple paths. Consequently, it exhibits a limited ETG compared with the BB and RH methods, particularly under low-noise conditions where the full decoding capacity is rarely required.

In this work, we propose a novel quasi-optimal path convergence (QOPC)-aided ET technique for parallel AE-SC decoding, which achieves an enhanced ETG compared with the PMT-aided ET method. The major contributions of this study are summarized as follows:

- We present a concise proof that PM convergence (PMC) arises in AE-SC decoding when multiple SC constituent decoders recover the codeword. Additionally, we analyze the occurrence of a zero path penalty (ZPP) in the PPM trajectory of SC decoding based on channel polarization theory. With PMC and ZPP establishing the theoretical foundation, we explore a QOPC phenomenon, which characterizes a PPM convergence feature in AE-SC decoding.
- Observing that a strong QOPC indicates high confidence in the involved paths being ultimately optimal, we propose a novel QOPC-aided ET technique that leverages sufficiently intense QOPC to reliably identify the best SC decoding path at runtime. The QOPC-aided ET method relies on implementation-friendly threshold comparisons with two simple parameters, which are appropriately optimized to balance the ETG and performance degradation. Once the QOPC-aided ET criterion is satisfied during the AE-SC decoding, only the identified path proceeds to complete decoding, while the others are terminated early.
- The block error rate (BLER) and the ETG of the proposed QOPC-aided AE-SC (QOPCA-AE-SC) decoder are extensively evaluated across multiple RM codes with various lengths and rates. The numerical results demonstrate that under fully parallel AE-SC decoding, the proposed QOPC-aided ET method incurred a minimal BLER loss and consistently outperformed the baseline PMT-aided ET method in terms of the ETG at a realistic target BLER of $10^{-3}$, with more pronounced improvements for higher-rate RM codes. Particularly, for a 256-length RM code with an information length of 219, the QOPC-aided ET method achieved a 47.4% reduction in the average complexity, while the PMT-aided ET method attained a minor complexity reduction of only 6.5%. Additionally, at a lower BLER that approached $10^{-5}$, the QOPC-aided ET method achieved a remarkable complexity reduction of 86.7% when applied to a partially parallel framework of AE-SC decoding.

The rest of this paper is organized as follows. Section 2 provides an overview of the RM codes, the AE-SC decoder, and the baseline PMT-aided ET method. Section 3 introduces the proposed QOPC-aided ET technique and its algorithmic implementation. Section 4 presents simulation results and comparisons with related works, along with discussions on the extensibility and practicality of the proposed method. Finally, Section 5 concludes this paper.

## 2. Preliminaries

### 2.1. Reed–Muller Codes

An RM code RM (*m*,*r*), specified by the integers *m* and *r*, is a linear block code with length $n=2^{m}$ and dimension k=∑i=0rmi. The generator matrix of RM (*m*,*r*) is derived from the Hadamard matrix $G_{n}={\left[\begin{array}{cc} 1 & 0\\ 1 & 1 \end{array}\right]}^{⊗m}$ by selecting rows with a Hamming weight of at least $2^{m-r}$, where (·)${⊗}^{m}$ denotes the *m*-th Kronecker power of a matrix. Similar to polar codes, the indices of selected rows can be defined as the information set $A=\{a_{0},a_{1},\ldots ,a_{k-1}\}$, while the remaining row indices form the frozen set $F=\{f_{0},f_{1},\ldots ,f_{n-k-1}\}$. An RM (*m*,*r*) codeword $x=[x_{0},x_{1},\cdots ,x_{n-1}]$ is generated by transforming a binary message vector $u=[u_{0},u_{1},\cdots ,u_{n-1}]$ into $x=uG_{n}$, where u is constructed by setting $u_{i}$ as an information bit if i∈A, and 0 otherwise.

### 2.2. Successive Cancellation Decoding

**Figure 1:**
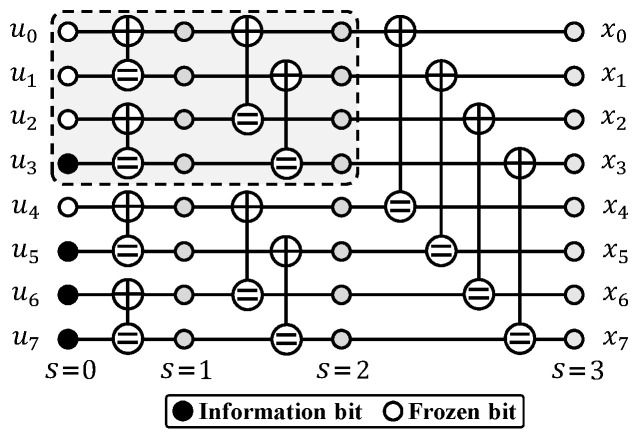
FFG representation of an RM(3,1) code. White and black circles at the 0-th stage represent frozen bits and information bits, respectively.

As illustrated in Figure 1, an RM (*m*,*r*) code can be represented by an (m+1)-stage Forney-style factor graph (FFG) [3]. In the FFG, the 0-th stage corresponds to the message vector u, while the *m*-th stage corresponds to the codeword x. The SC decoding can be efficiently implemented based on the FFG representation. Let $y^{s}$ and ${\hat{u}}^{s}$ denote the log-likelihood ratio (LLR) messages and the hard decisions at the *s*-th stage, respectively. Both $y^{s}$ and ${\hat{u}}^{s}$ are calculated recursively but in opposite directions on the FFG. The soft messages $y^{m}$ at the right-most stage are initialized using the channel LLRs $y^{ch}$, and then processed stage-by-stage from right to left with the following update rules:

(1) $$\begin{array}{cc} y_{i}^{s-1} & =f(y_{i}^{s},y_{i+2^{s-1}}^{s}), \end{array}$$

(2) $$\begin{array}{cc} y_{i+2^{s-1}}^{s-1} & =g(y_{i}^{s},y_{i+2^{s-1}}^{s},{\hat{u}}_{i}^{s-1}), \end{array}$$

where the subscript denotes the bit index, and the functions *f* and *g* are defined as

(3) $$\begin{array}{cc} f(a,b) & =\ln(\frac{e^{a+b}+1}{e^{a}+e^{b}}), \end{array}$$

(4) $$\begin{array}{cc} g(a,b,c) & =(1-2c)a+b. \end{array}$$

The recursion terminates at the 0-th stage, where either an information bit estimate is made or the frozen bit value 0 is returned. Then, the hard decisions are propagated from left to right as ${\hat{u}}_{i}^{s+1}={\hat{u}}_{i}^{s}⊕{\hat{u}}_{i+2^{s}}^{s}$ and ${\hat{u}}_{i+2^{s}}^{s+1}={\hat{u}}_{i+2^{s}}^{s}$.

### 2.3. Automorphism Ensemble Decoding

An automorphism π of an *n*-length code C is a permutation on the set {0,1,⋯,n−1} that satisfies π(x)∈C for all x∈C, where π(x) results in the codeword $x^{\prime }$ with components rearranged as $x_{i}^{\prime }=x_{\pi (i)}$ [11]. In other words, every codeword is mapped to another codeword of the same code. The automorphism group Aut(C) of a code C is the set of all automorphisms of the code. For RM codes, the automorphism group is known to be the general affine group GA(m) [12], which consists of all affine bijections over $F_{2}^{m}$. An affine bijection is defined as the mapping $z^{\prime }=Az+b$, where $A\in F_{2}^{m\times m}$ is an invertible matrix, and $b\in F_{2}^{m\times 1}$ is an arbitrary vector. Here, $z^{\prime }$ and z are the *m*-bit binary representations of the code bit positions *i* and π(i), respectively.

**Figure 2:**
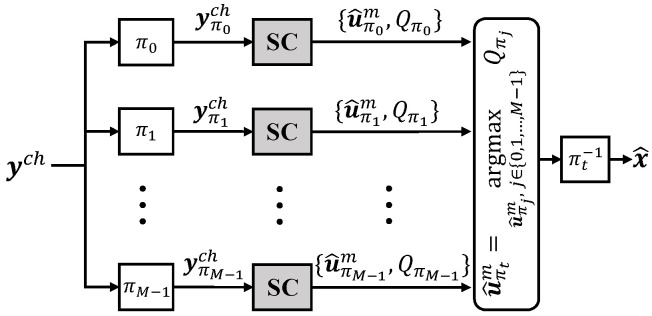
Framework of the AE-SC-*M* decoder.

Figure 2 illustrates the framework of an AE-SC-*M* decoder, which utilizes *M* randomly sampled permutations from the automorphism group of RM codes, with each one denoted as $\pi_{j}$ for j∈{0,1,2,⋯,M−1}. According to each permutation $\pi_{j}$, the channel LLRs $y^{ch}$ are interleaved with $y_{\pi_{j}}^{ch}$, which is then decoded by an SC decoder $D_{\pi_{j}}^{\mathrm{SC}}$. Based on a least-squares metric [11] or the LLR-based PM [19], the optimal candidate is selected from the *M* codeword estimates. In this study, we considered the whole automorphism group of RM codes to generate permutation samples. Additionally, we used the LLR-based PM to select the output codeword estimate, which is defined as

(5) $$Q_{\pi_{j}}=\sum_{i=0}^{n-k-1}q_{f_{i},\pi_{j}}=\sum_{i=0}^{n-k-1}\min\left\{0,\left(1-2{\hat{u}}_{f_{i},\pi_{j}}^{0}\right)y_{f_{i},\pi_{j}}^{0}\right\},$$

where $q_{f_{i},\pi_{j}}$ denotes the penalty of the PM [19] when the path $D_{\pi_{j}}^{\mathrm{SC}}$ decodes its *i*-th frozen bit. Equation (5) suggests that the LLR-based PM is monotonic in the SC decoding and allows for determining the optimal path in AE-SC decoding once the last frozen bit is decoded by all the SC constituent decoders. Let $\pi_{t}$ denote the permutation that delivers the best (largest) PM $Q_{\pi_{t}}$. The AE-SC-*M* decoder produces the output codeword estimate $\hat{x}$ by deinterleaving ${\hat{u}}_{\pi_{t}}^{m}$ with $\pi_{t}$.

### 2.4. PMT-Aided Early Termination for AE-SC Decoding

It has been proven in [10] that Equation (5) can be rewritten as

(6) $$Q_{\pi_{j}}=\sum_{i=0}^{n-1}\min\left\{0,\left(1-2{\hat{u}}_{i,\pi_{j}}^{m}\right)y_{i,\pi_{j}}^{m}\right\}.$$

Without loss of generality, we assumed that an *n*-length all-zero codeword is transmitted over a binary input additive white Gaussian noise (BI-AWGN) channel with noise variance $\sigma^{2}$ using binary phase-shift keying (BPSK) modulation. If the noisy codeword is recovered, Equation (6) is further expressed as

(7) $$Q_{\pi_{j}}=\sum_{i=0}^{n-1}\min\left\{0,y_{i,\pi_{j}}^{m}\right\}.$$

As each LLR $y_{i,\pi_{j}}^{m}$ follows the normal distribution $N(\frac{2}{\sigma^{2}},\frac{4}{\sigma^{2}})$ [15], each element of Equation (7) is sampled from a random variable with the cumulative distribution function (CDF):

(8) $$\tilde{F}\left(x\right)=\left\{\begin{array}{cc} F(x), & \mathrm{if}\;x<0,\\ 1, & \mathrm{otherwise}, \end{array}\right.$$

where *F* denotes the CDF of $N(\frac{2}{\sigma^{2}},\frac{4}{\sigma^{2}})$. Using Equation (8) and the central limit theorem, the CDF of Equation (7), denoted as $F_{Q}$, can be approximated by the CDF of $N(n\tilde{\mu },n{\tilde{\sigma }}^{2})$, where $\tilde{\mu }$ and ${\tilde{\sigma }}^{2}$ are the mean and variance of the distribution with the CDF defined by Equation (8), respectively. Based on $F_{Q}$, the PMT-aided ET method [10] evaluates a PM threshold *T* with a probability $P_{T}$, where $T=F_{Q}^{-1}\left(P_{T}\right)$. $P_{T}$ is typically set to a small value to ensure a low probability that the PM of the correct SC decoding path exceeds *T*. If the partial PM of any SC decoding path falls below *T* in the AE-SC decoding at runtime, that path can be terminated. The authors in [10] indicate that for the PMT-aided AE-SC (PMTA-AE-SC) decoder, its BLER at the SNR used for PM threshold evaluation is lower-bounded by $P_{T}$.

When the LLR-based PM [19] is used to determine the final output codeword estimate (or path), the PMT-aided ET method incurs no performance degradation compared with the original AE-SC decoding. As the PMT-aided ET technique terminates SC decoding paths whose partial PMs exceed a predefined threshold *T* at runtime, the monotonicity of PM updating guarantees that the best PM of the paths that are not terminated early must be the best PM of all paths. In cases where all SC decoding paths are terminated during the AE-SC decoding process, all paths are allowed to resume from the termination point to complete the decoding. The final output path is then determined in the same manner as in the original AE-SC decoding. Therefore, whether or not any paths survive the PMT-aided ET criterion, the path with the best PM is invariably selected as the final output. This mechanism ensures that the BLER performance of the PMTA-AE-SC decoder is strictly identical to that of the original AE-SC decoder.

## 3. Quasi-Optimal Path Convergence-Aided AE-SC Decoding

In this section, by jointly investigating the final PM diversity and the partial PM trajectory of the SC constituent decoders in the AE-SC decoding, we develop a QOPC-aided ET technique to reduce the AE-SC decoding complexity by reliably identifying the optimal SC decoding path early.

### 3.1. Analysis of the Path Metric in the AE-SC Decoding

#### 3.1.1. Final PM Diversity

It has been shown that the AE-SC-*M* decoder may produce fewer than *M* unique codeword estimates [10], indicating that multiple SC decoding paths could share the decoding results. Inspired by this, we investigated the relationship between the PMs of the SC constituent decoders that have the same codeword estimate.

**Proposition 1**. *If two SC constituent decoders $D_{\pi_{\alpha }}^{\mathrm{SC}}$ and $D_{\pi_{\beta }}^{\mathrm{SC}}$ generate the same codeword estimate in the AE-SC decoding, they also have an identical PM.*

**Proof of Proposition 1**. To prove this proposition, we rewrite the Equation (6) in a vector form as

(9) $$Q_{\pi_{j}}=\vec{\sum }\vec{\min}\left\{0,\left(1-2\cdot {\hat{u}}_{\pi_{j}}^{m}\right)\cdot y_{\pi_{j}}^{ch}\right\},$$

where the operations are applied element-wise, and 0 and 1 represent all-zero and all-one vectors, respectively. Then, by deinterleaving the codeword estimate ${\hat{u}}_{\pi_{j}}^{m}$ and the input $y_{\pi_{j}}^{ch}$ of $D_{\pi_{j}}^{\mathrm{SC}}$ with permutation $\pi_{j}$, Equation (9) is transformed into

(10) $$\begin{array}{cc} Q_{\pi_{j}} & =\vec{\sum }\vec{\min}\left\{0,\left(1-2\cdot \pi_{j}^{-1}\left({\hat{u}}_{\pi_{j}}^{m}\right)\right)\cdot y^{ch}\right\}. \end{array}$$

As $\pi_{\alpha }^{-1}\left({\hat{u}}_{\pi_{\alpha }}^{m}\right)=\pi_{\beta }^{-1}\left({\hat{u}}_{\pi_{\beta }}^{m}\right)$, we conclude that $Q_{\pi_{\alpha }}=Q_{\pi_{\beta }}$.    □

Intuitively, as the SNR increases, more SC decoders in the AE-SC decoding can successfully decode the corrupted codeword, converging to the same PM, as stated in Proposition 1. Consequently, a lower noise power results in a lower PM diversity in the AE-SC decoding because more SC decoding paths are involved in the PM convergence (PMC).

#### 3.1.2. Partial PM Trajectory

Considering RM codes from a polar codes point of view, the SNR also impacts the error probability of synthesized subchannels according to the channel polarization theory [3]. For the BI-AWGN channel, the error probability of the *i*-th subchannel, denoted as $H_{i}$, can be evaluated via the Gaussian approximation (GA) [21] as

(11) $$H_{i}\approx \frac{1}{2}erfc\left(\sqrt{E[y_{i}^{0}]/4}\right),$$

where $er\;fc\left(x\right)=\left(2/\sqrt{\pi }\right)\int_{x}^{\infty }e^{-\eta^{2}}d\eta$ and $E[y_{i}^{0}]$ is the mean of $y_{i}^{0}$. Equation (5) suggests that the PM of SC decoding accumulates only when a frozen bit is decoded with a negative soft decision. Thus, if the $f_{i}$-th subchannel becomes more reliable (i.e., a lower $H_{f_{i}}$) due to an increased SNR [15], the *i*-th frozen bit is more likely to be correctly soft-decoded by the SC decoder, thereby reducing the probability of penalizing the PM. As a result, the PM tends to remain unchanged at the *i*-th frozen bit, which increases the frequency of observing a zero path penalty (ZPP) [19] at runtime.

### 3.2. Quasi-Optimal Path Convergence-Aided Early Termination

Let $Q_{f_{i},\pi_{j}}$ and $Q_{f_{i}}^{o}$ denote the PPM of the decoding path $D_{\pi_{j}}^{\mathrm{SC}}$ and the optimal PPM among the *M* paths after the *i*-th frozen bit is decoded, respectively, where 0≤i<n−k and 0≤j<M. Specifically, $Q_{f_{i},\pi_{j}}=\sum_{d=0}^{i}q_{f_{d},\pi_{j}}$ and $Q_{f_{i}}^{o}=\arg\max Q_{f_{i},\pi_{j}}$ for j∈{0,1,…,M−1}. Furthermore, let $Q_{f_{i}}^{\rho }$ denote the mode among the *M* PPMs after the *i*-th frozen bit is decoded, which is shared by ρ paths. Since $Q_{f_{i}}^{\rho }$ does not necessarily equal the currently optimal PPM $Q_{f_{i}}^{o}$, we refer to the paths sharing $Q_{f_{i}}^{\rho }$ as quasi-optimal paths, and define this PPM convergence phenomenon among the quasi-optimal paths as (*i*,ρ)-quasi-optimal path convergence (QOPC).

**Figure 3:**
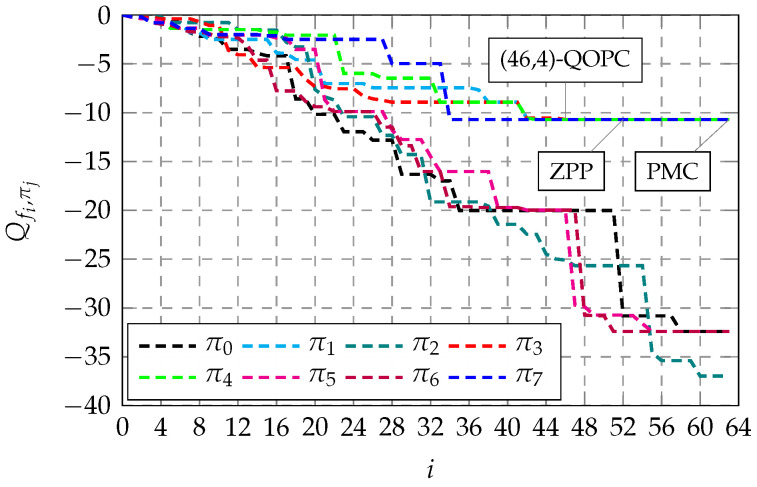
Sampled partial PM trajectories in an AE-SC-8 decoder for decoding an RM(7,3) codeword at an SNR of 2.5 dB.

The aforementioned PMC and ZPP jointly enable the occurrence of QOPC. In particular, if ρ paths share the best PM $Q_{\pi_{t}}$ in the PMC by correctly decoding the codeword *and* exhibit the ZPP on the last n−k−1−i frozen bits, an (*i*,ρ)-QOPC exists if $Q_{\pi_{t}}$ is the mode among the *M* PPMs after the *i*-th frozen bit is decoded. Figure 3 illustrates the PPM trajectories in an AE-SC-8 decoder for decoding an RM(7,3) codeword at an SNR of 2.5 dB, where four SC decoding paths recover the codeword. It can be observed that a (46,4)-QOPC occurs due to the presence of the PMC and ZPP. If such a QOPC can be accurately and timely detected in AE-SC decoding, we can allow only one involved path to proceed while terminating the rest of the M−1 paths early, which reduces the overall decoding complexity without degrading the error-correction performance.

Note that *i* and ρ represent the occurrence position and the convergence intensity of the QOPC, respectively. Both *i* and ρ vary with the SNR, as the SNR influences the intensity of the final PMC and the probability of the runtime ZPP at frozen bits. Based on this observation, we propose utilizing a pair of integer parameters (λ,ω), with 0≤λ<n−k−1 and 1<ω≤M, to detect the QOPC for early termination in the AE-SC decoder. Specifically, the proposed QOPC-aided ET method operates as follows: when the *i*-th frozen bit is decoded in the parallel AE-SC decoding, where λ≤i<n−k−1, the ρ indices of the SC decoding paths that share the PPM mode $Q_{f_{i}}^{\rho }$ form a candidate decoder index set $S_{Q_{f_{i}}^{\rho }}=\left\{J_{0},J_{1},\cdots ,J_{\rho -1}\right\}\subseteq \left\{0,1,\cdots ,M-1\right\}$, which is arranged in ascending order (if there is more than one mode among the *M* PPMs after the *i*-th frozen bit is decoded, the QOPC-aided ET verification will be skipped at this frozen bit, and the AE-SC decoding continues). If ρ≥ω, only the path with the lowest index in $S_{Q_{f_{i}}^{\rho }}$, denoted as $D_{\pi_{J_{0}},Q_{f_{i}}^{\rho }}^{\mathrm{SC}}$, proceeds to generate its complete codeword estimate as the final output of the AE-SC decoding, while the rest of the M−1 paths are terminated. If the QOPC-aided ET criterion is never met, the proposed QOPCA-AE-SC decoder reduces to the original AE-SC decoder. The formal description of the QOPC-aided ET technique is given in Algorithm 1.

**Algorithm 1:**
QOPCA-AE-SC-*M* Decoding Algorithm


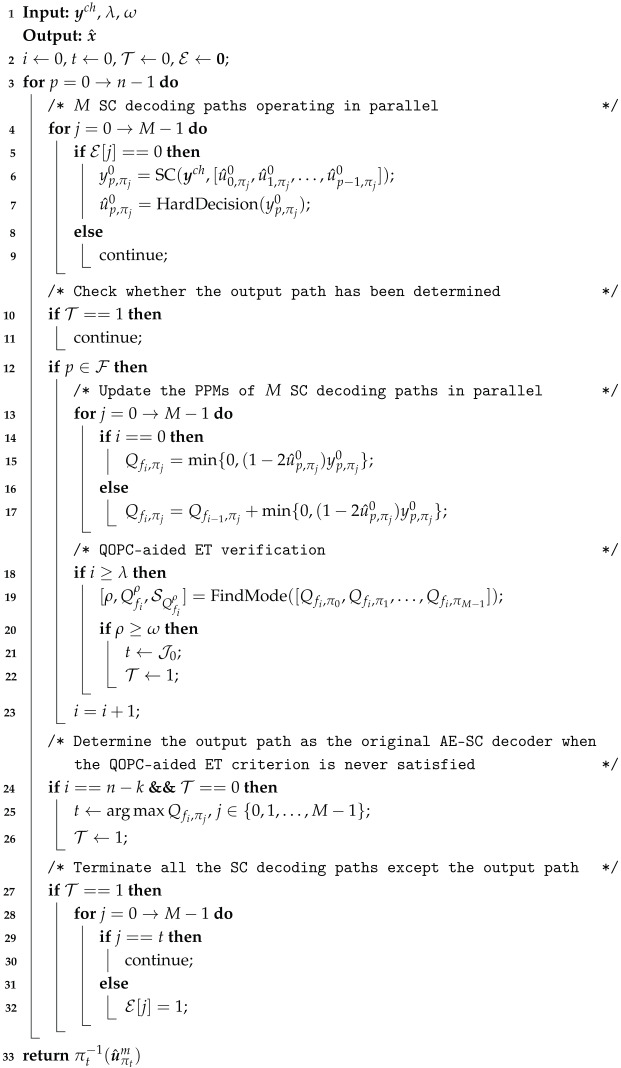



### 3.3. Complexity and Performance of QOPCA-AE-SC Decoding

#### 3.3.1. Complexity

Assuming that an (*i*,ρ)-QOPC satisfies the proposed QOPC-aided ET criterion during the AE-SC decoding, the decoding complexity of this RM codeword, denoted as $C_{i}^{\mathrm{QOPC}}$, mainly results from three parts: one complete SC decoding corresponding to the selected output path, M−1 unfinished SC decodings terminated at the *i*-th frozen bit, and i−λ+1 ET verifications in total. Let $O_{p}^{\mathrm{SC}}$ represent the number of *f* and *g* operations required to decode ${\hat{u}}_{p}^{0}$ in a non-parallelized SC decoding implementation, where 0≤p<n.

The complexity of one complete SC decoding can be expressed as $O_{n-1}^{\mathrm{SC}}$. The complexity of an incomplete SC decoding, which is terminated after the *i*-th frozen bit is decoded, corresponds to $O_{f_{i}}^{\mathrm{SC}}$. Regarding the complexity of a single ET verification, it primarily involves determining $Q_{f_{i}}^{\rho }$ and its associated path indices. In this work, a hash table is employed to identify the mode among *M* PPMs after the *i*-th frozen bit is decoded. Initially, the *M* PPMs are traversed to compute the frequency of each PPM and record their indices, resulting in a complexity of *M*. Let $B_{i}$ denote the number of unique elements among the *M* PPMs, where $B_{i}\leq M$. Subsequently, the hash table, with a size of $B_{i}$, is traversed to find the mode $Q_{f_{i}}^{\rho }$, along with its frequency ρ and associated path indices $S_{Q_{f_{i}}^{\rho }}$, incurring a complexity of $B_{i}$. Therefore, each ET verification requires a total complexity of $M+B_{i}$. Accordingly, $C_{i}^{\mathrm{QOPC}}$ is expressed as

(12) $$C_{i}^{\mathrm{QOPC}}=O_{n-1}^{\mathrm{SC}}+O_{f_{i}}^{\mathrm{SC}}\cdot \left(M-1\right)+\sum_{d=\lambda }^{i}\left(M+B_{d}\right).$$

Equation (12) demonstrates that $C_{i}^{\mathrm{QOPC}}$ increases monotonically with *i*. Consequently, the worst-case complexity of the proposed QOPCA-AE-SC decoder, denoted as $C_{\max}^{\mathrm{QOPC}}$, corresponds to the scenario where the QOPC-aided ET criterion is never satisfied. In this case, the output SC decoding path is determined when all the n−k frozen bits are decoded, where all paths generate the final PM. Only the selected output path is required to continue decoding the remaining information bits, while the rest of the M−1 paths can be terminated. Additionally, a single ET verification exhibits its worst-case complexity when $B_{i}=M$. Therefore, $C_{\max}^{\mathrm{QOPC}}$ is computed using

(13) $$C_{\max}^{\mathrm{QOPC}}=O_{n-1}^{\mathrm{SC}}+O_{f_{n-k-1}}^{\mathrm{SC}}\cdot \left(M-1\right)+\sum_{d=\lambda }^{n-k-1}2M.$$

In contrast, the best-case complexity of the proposed QOPCA-AE-SC decoder, denoted as $C_{\min}^{\mathrm{QOPC}}$, arises if the QOPC-aided ET criterion is satisfied at the λ-th frozen bit with all PPMs being the same, which is given by

(14) $$C_{\min}^{\mathrm{QOPC}}=O_{n-1}^{\mathrm{SC}}+O_{f_{\lambda }}^{\mathrm{SC}}\cdot \left(M-1\right)+M+1.$$

Obviously, the average complexity of the QOPCA-AE-SC decoder, denoted as $C^{\mathrm{QOPC}}$, is bounded by $C_{\max}^{\mathrm{QOPC}}$ and $C_{\min}^{\mathrm{QOPC}}$. At relatively low SNR levels, $C^{\mathrm{QOPC}}$ approaches $C_{\max}^{\mathrm{QOPC}}$ since the QOPC with a convergence intensity exceeding the threshold ω rarely occurs. Conversely, as SNR increases, $C^{\mathrm{QOPC}}$ approaches $C_{\min}^{\mathrm{QOPC}}$ due to the increased probability of detecting a sufficiently strong QOPC at an early stage of AE-SC decoding. Therefore, when evaluating $C^{\mathrm{QOPC}}$ within the operating range of certain RM codes, it is expected to exhibit a monotonically decreasing trend as SNR increases.

Equations (13) and (14) indicate that the parameter λ in the proposed QOPC-aided ET criterion, which marks the starting frozen bit for the QOPC detection, determines both the maximum complexity overhead from the ET verification and the minimum decoding complexity. To select a λ that balances both considerations, we propose a universal single-recursion upper sub-FFG (SRUS) rule for RM codes, where the λ-th frozen bit is the last frozen bit in the SRUS of the code. Figure 1 illustrates the SRUS of the RM(3,1) code, which is highlighted with a dotted line box. This SRUS rule provides an efficient trade-off between the benefits of ET and the additional ET verification cost. In essence, the SRUS is equivalent to the largest left subtree of the RM code tree. Thus, the selected λ allows for a significant complexity reduction if the QOPC-aided ET criterion is satisfied at the λ-th frozen bit, enabling the skipping of nearly half of the AE-SC decoding computations. Additionally, according to the row-weight-based frozen set formulation criterion of the RM codes, no more than half of the frozen bits will be involved in the QOPC detection, thereby minimizing the worst-case complexity overhead from the QOPC-based ET verifications.

For comparison, the complexity of the original AE-SC decoder, denoted as $C^{\mathrm{OG}}$, can also be divided into three components: one complete SC decoding for the output path, M−1 unfinished SC decodings terminated after decoding all the frozen bits, and M−1 comparisons among the *M* PMs to determine the output path. Thus, $C^{\mathrm{OG}}$, which does not vary with the noise level, is calculated using

(15) $$C^{\mathrm{OG}}=O_{n-1}^{\mathrm{SC}}+O_{f_{n-k-1}}^{\mathrm{SC}}\cdot \left(M-1\right)+M-1.$$

Regarding the complexity description of the baseline PMTA-AE-SC decoder for decoding a single RM codeword, we define an *M*-length vector $v=[v_{0},v_{1},\cdots ,v_{M-1}]$, where $v_{j}$, with 0≤j<M, denotes the number of comparisons between the PPM $Q_{f_{i},\pi_{j}}$ and the threshold *T* in the path $D_{\pi_{j}}^{\mathrm{SC}}$. Let $C_{v}^{\mathrm{PMT}}$ denote the AE-SC decoding complexity of this codeword when using the PMT-aided ET method. It is worth noting that the threshold comparison is conducted only when the path $D_{\pi_{j}}^{\mathrm{SC}}$, which is not terminated, updates its PPM $Q_{f_{i},\pi_{j}}$ by decoding a frozen bit. Thus, we have $0<v_{j}\leq n-k$. If the path $D_{\pi_{j}}^{\mathrm{SC}}$ survives the PMT-based ET verification at all frozen bits, it exhibits the final $v_{j}=n-k$. As analyzed in Section 2.4, the PMTA-AE-SC decoder shares the output path with the original AE-SC decoder, incurring no performance loss. Therefore, in the PMTA-AE-SC decoder, only the output path $D_{\pi_{t}}^{\mathrm{SC}}$ with the optimal PM $Q_{\pi_{t}}$ proceeds to decode the remaining information bits after the last frozen bit is decoded. If the path $D_{\pi_{j}}^{\mathrm{SC}}$ is terminated after $v_{j}$ threshold comparisons, its SC decoding process stops after the $\left(v_{j}-1\right)$-th frozen bit is decoded. Similarly, $C_{v}^{\mathrm{PMT}}$ can be divided into three parts: one complete SC decoding corresponding to the best path, M−1 unfinished SC decodings, and threshold comparisons for the PMT-based ET verification. Then, $C_{v}^{\mathrm{PMT}}$ can be expressed as

(16) $$C_{v}^{\mathrm{PMT}}=O_{n-1}^{\mathrm{SC}}+\sum_{j\in \{0,1,\cdots ,M-1\}/t}O_{f_{v_{j}-1}}^{\mathrm{SC}}+\sum_{j=0}^{M-1}v_{j}.$$

By comparing Equations (12), (15) and (16), we observed that the ETG of the baseline PMT-aided ET method primarily stems from a small portion of paths not being early terminated. Specifically, the PMTA-AE-SC decoder achieves its peak ETG in the low-to-medium SNR regions where the BLER is high. This is because in the high-SNR region, most paths can recover the corrupted codeword and typically do not produce a PM that exceeds the threshold *T*, which makes the PMT-aided ET method less effective and incurs a limited ETG. For the proposed QOPC-aided ET method, a moderate ETG can be observed in the low-SNR regions because the QOPC-based ET criterion is rarely met when a reliable threshold ω is adopted. However, as the SNR increases, the QOPC-based ET criterion tends to be satisfied at an earlier stage of AE-SC decoding, which significantly reduces the complexity of the unfinished SC decoding paths, thus exhibiting a substantial ETG. Therefore, compared with the baseline PMTA-AE-SC decoder, the proposed QOPCA-AE-SC decoder begins to demonstrate a notable ETG improvement starting from the medium SNR region, and the gain progressively grows as the SNR increases.

#### 3.3.2. Performance

The proposed QOPC-aided ET technique was designed to reliably identify the SC decoding paths that will ultimately recover the codeword at runtime. The QOPC phenomenon can be viewed as a pre-convergence of these final optimal paths. As the AE-SC decoding progresses, the intensity of the QOPC will increase since the partial PM trajectories tend to be converged. Therefore, the parameter ω, as the QOPC intensity threshold in our ET method, dominates the probability of the selected path indeed being optimal. On the one **hand**, **if** ω is too small, the detected QOPC may provide limited confidence that the path $D_{\pi_{J_{0}},Q_{f_{i}}^{\rho }}^{\mathrm{SC}}$ is equivalent to the output path of the original AE-SC decoder, potentially leading to a performance loss. On the other hand, if ω is too large, although the performance degradation is negligible, the complexity reduction will also be compromised.

**Figure 4:**
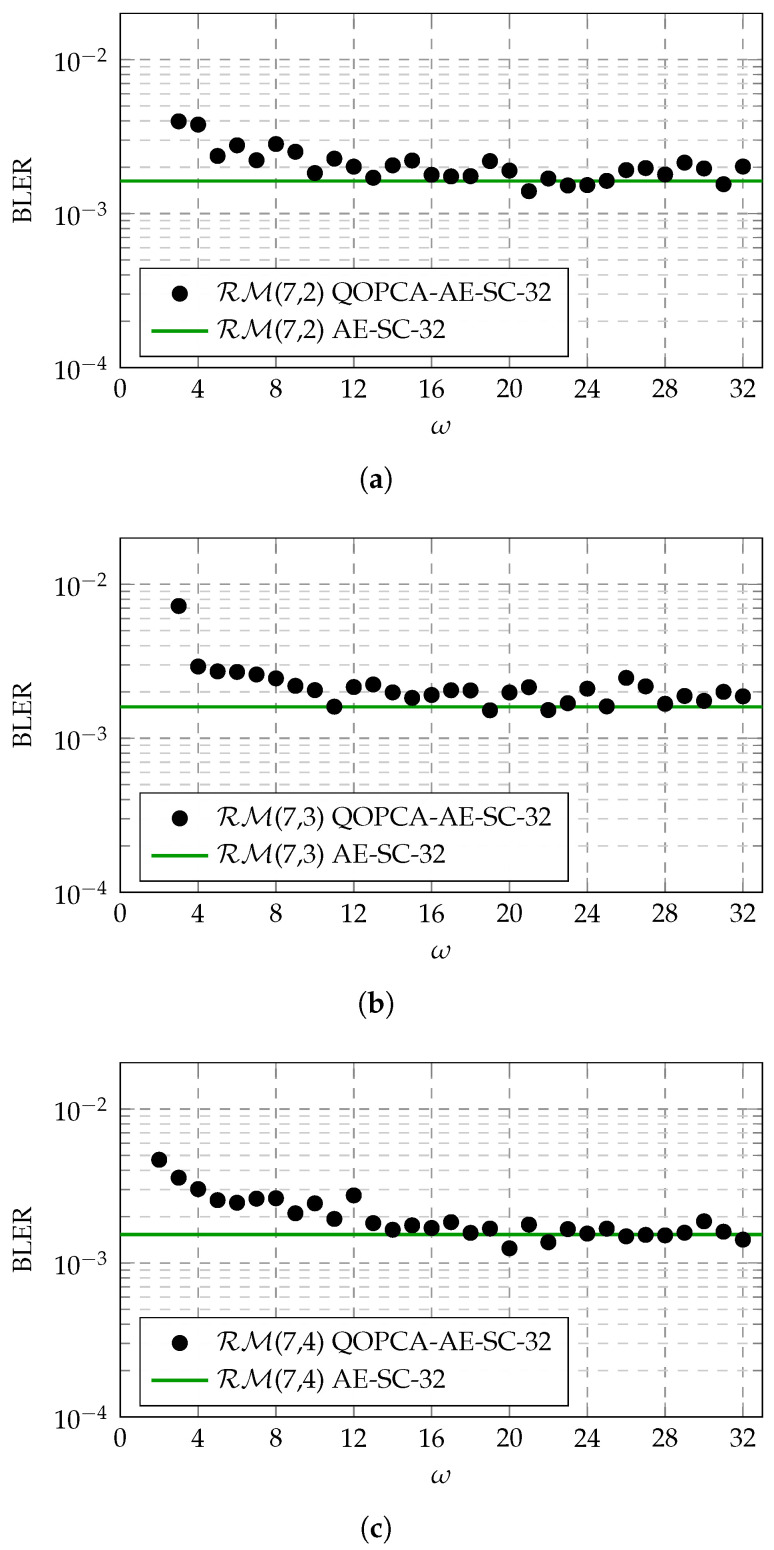
BLER variation against various ω values used in the QOPCA-AE-SC-32 decoder for the RM(7,2), RM(7,3), and RM(7,4) codes at specific operating SNR points, where the original AE-SC decoder achieved a BLER of $1.6\times 10^{-3}$. (**a**) BLER of the QOPCA-AE-SC-32 decoder with various ω values for the RM(7,2) code at an SNR of −0.9 dB. (**b**) BLER of the QOPCA-AE-SC-32 decoder with various ω values for the RM(7,3) code at an SNR of 3.0 dB. (**c**) BLER of the QOPCA-AE-SC-32 decoder with various ω values for the RM(7,4) code at an SNR of 6.0 dB.

In this work, we propose evaluating the threshold ω of the proposed QOPC-based ET criterion at a target BLER of around $10^{-3}$, which is near the realistic working point for many wireless applications [20]. Figure 4 demonstrates the ω evaluation results for the QOPCA-AE-SC-32 decoder applied to the RM(7,2), RM(7,3), and RM(7,4) codes, where λ=56, 41, and 21, respectively, according to the aforementioned SRUS rule, and the target BLER achieved by the original AE-SC decoder was $1.6\times 10^{-3}$. It can be seen that as ω increased, the BLER loss introduced by our QOPC-aided ET method decreased, where ω values larger than 8 exhibited minimal loss for all three RM codes.

To maximize the ETG of the proposed QOPC-aided ET method, it is intuitive to configure ω as the smallest integer that does not incur undue loss for certain RM codes at a target SNR, which maximizes the probability of satisfying the QOPC-based ET criterion. However, the ω threshold evaluation above serves only as a quick and preliminary reference. We need to assess that the selected ω does not introduce excessive losses at higher SNR points. Additionally, for different RM codes with the same length, it is preferable to unify the ω values in decoder implementations. Therefore, based on the ω evaluation examples depicted in Figure 4, we propose selecting a universal ω=16 for the considered RM codes, which offers sufficient confidence in identifying the best path early when the QOPC-based ET criterion is met across a wide range of SNRs. Notably, this ω selection process can be conducted offline, enabling the optimized (λ,ω) configurations to be preset for practical AE-SC decoder implementations.

## 4. Simulation Results and Discussions

In this section, we describe a comprehensive evaluation of the proposed QOPCA-AE-SC decoder, which involved examining its performance across multiple RM codes with varying lengths and code rates. Specifically, three 128-length RM codes RM(7,2), RM(7,3), and RM(7,4) and three 256-length RM codes RM(8,3), RM(8,4), and RM(8,5) were considered in our experiments, which covered code rates from low to high. The automorphism ensemble size *M* for the AE-SC decoding was set to 32 and 256 for the 128-length RM codes and 256-length RM codes, respectively. The numerical results were obtained over the BI-AWGN channel that utilized BPSK modulation. Employing the optimized (λ, ω) configurations, the proposed QOPCA-AE-SC decoder was benchmarked against the baseline PMTA-AE-SC decoder [10], with a focus on the BLER and ETG metrics. According to the ω optimization strategy in Section 3.3.2, ω=32 is selected in the QOPCA-AE-SC-256 decoder for the three 256-length RM codes. For the PMTA-AE-SC decoder, we adopted $P_{T}=5\times 10^{-4}$ from [10] to evaluate the PM threshold *T* at a fixed SNR for each RM code. Table 1 outlines the parameters of the tested RM codes, along with the configurations utilized for our QOPCA-AE-SC decoder and the PMTA-AE-SC decoder.

### 4.1. Error-Correction Performance

This study focused on low-latency scenarios with small block sizes, which are commonly employed in control channels. In practical applications, control channels typically require low target BLER levels (e.g., $10^{-5}$ or lower) to ensure both a low latency and a low failure rate. To address this, we conduct extensive simulations to achieve BLER values approaching $10^{-5}$ and beyond. To comprehensively evaluate the error-correction performance of the proposed QOPCA-AE-SC decoder, we compared it with three benchmark decoders: the original AE-SC decoder [11], the PMTA-AE-SC decoder [10], and the SCL decoder [19]. The list size *L* of the SCL decoder was set equal to the automorphism ensemble size *M* of the AE-SC decoders in the experiments. For reference, the ML lower bound (MLLB) [16] for the considered RM codes, which was measured using the AE-SC decoder, was also plotted. Additionally, the BLER of a partially parallel adaptive QOPCA-AE-SC (PPA-QOPCA-AE-SC) decoder, which is thoroughly introduced in Section 4.3.2, was simulated to verify its effectiveness. In general, the PPA-QOPCA-AE-SC decoder is developed by applying the proposed QOPC-based ET method to the partially parallel AE-SC decoding framework. It incorporates a parameter θ, where 0<θ≤1, for adaptive parallelism control, enabling a more aggressive complexity reduction compared with the fully parallel QOPCA-AE-SC decoder.

**Figure 5:**
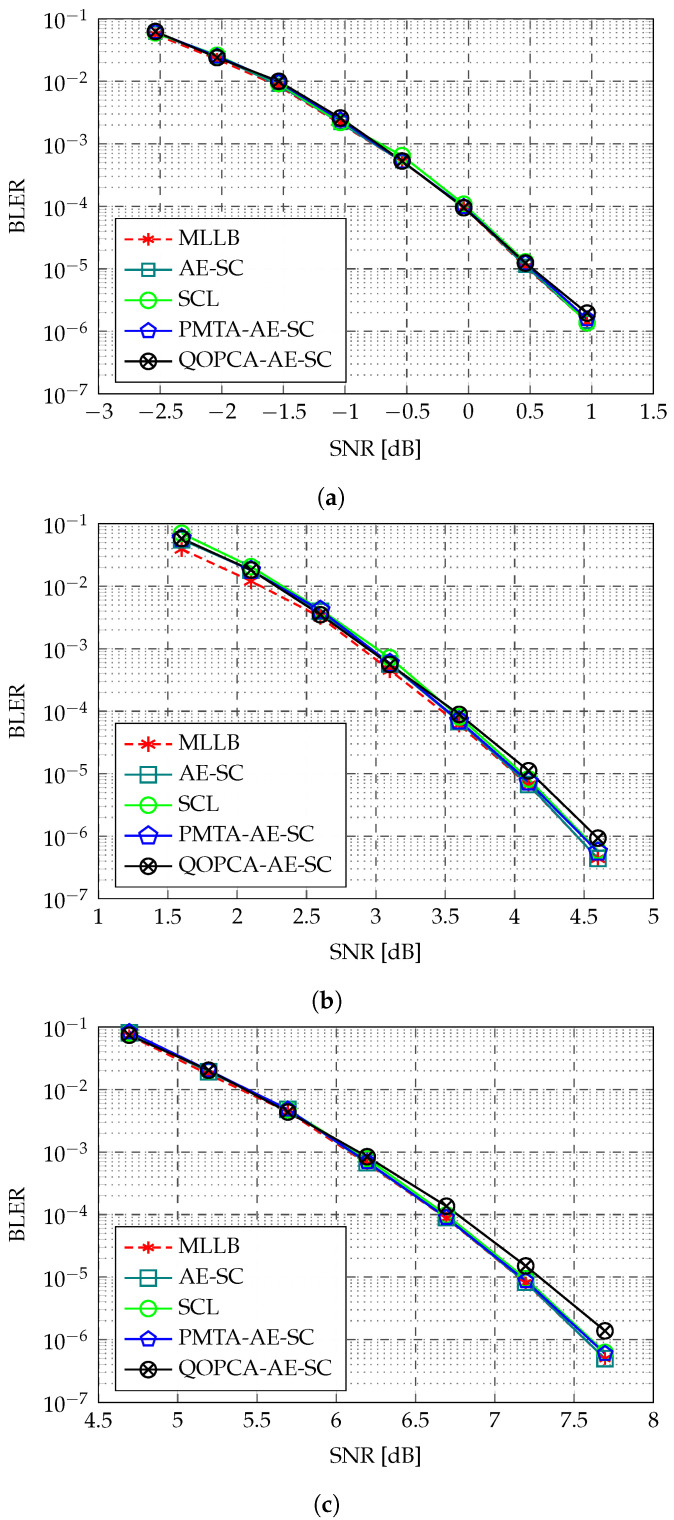
BLER comparison of MLLB [16], SCL decoder [19], AE-SC decoder [11], PMTA-AE-SC decoder [10], and our QOPCA-AE-SC decoder for the RM(7,2), RM(7,3), and RM(7,4) codes. (**a**) RM(7,2), n=128, k=29, and code rate = 0.23. (**b**) RM(7,3), n=128, k=64, and code rate = 0.50. (**c**) RM(7,4), n=128, k=99, and code rate = 0.77.

Figure 5 illustrates the BLER performance of various decoders for the three 128-length RM codes RM(7,2), RM(7,3), and RM(7,4). For these RM codes, the AE-SC-32 and SCL-32 decoders exhibited nearly identical BLERs, where both demonstrated near-ML performances with negligible losses to the MLLB at a BLER of $10^{-5}$. Moreover, the PMTA-AE-SC-32 decoder exhibited no BLER degradation compared with the AE-SC-32 decoder, which validated our performance analysis of the PMTA-AE-SC decoder in Section 2.4. At a BLER of $10^{-5}$, the proposed QOPCA-AE-SC-32 decoder showed no visible BLER loss to the AE-SC-32 decoder for the RM(7,2) code, while showing minimal losses of 0.09 dB and 0.13 dB for the RM(7,3) and RM(7,4) codes, respectively. Therefore, for all the considered 128-length RM codes, the proposed QOPCA-AE-SC-32 decoder maintained a near-ML performance for the AE-SC decoding and achieved a BLER comparable with the baseline PMTA-AE-SC-32 decoder.

**Figure 6:**
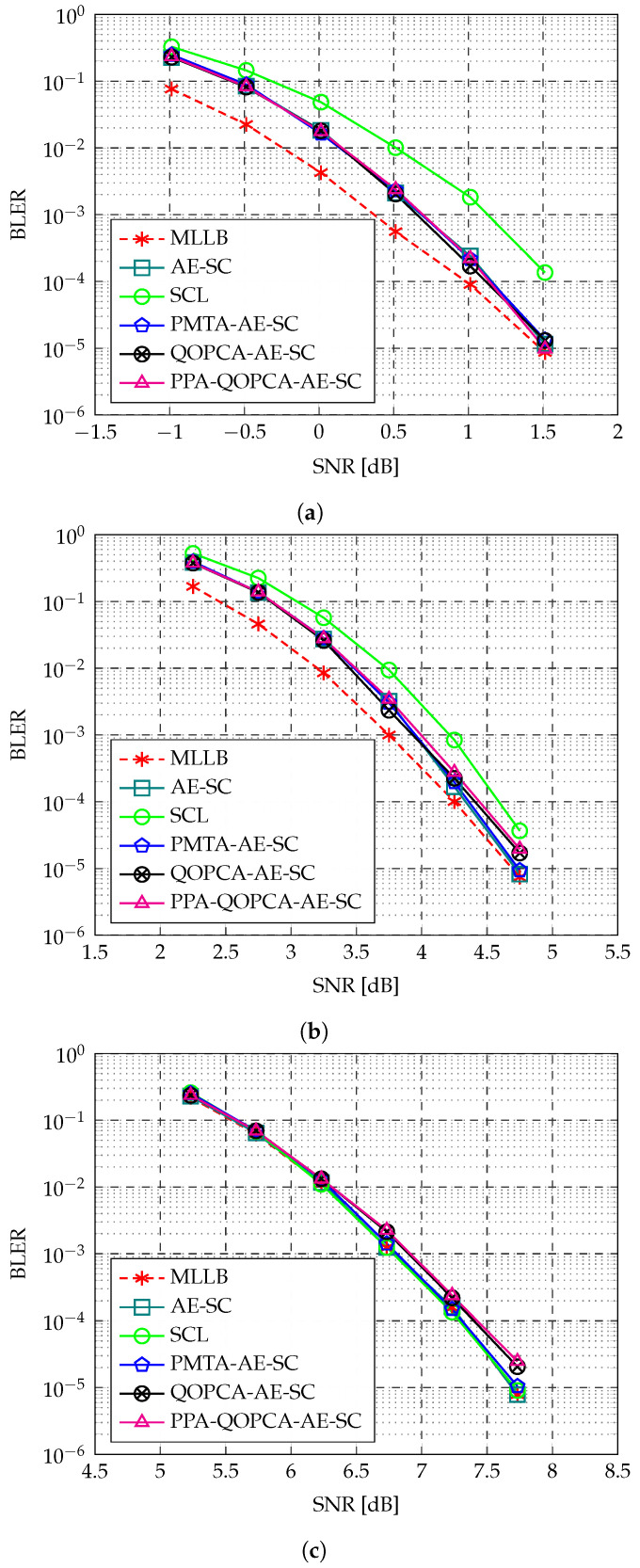
BLER comparison of MLLB [16], SCL decoder [19], AE-SC decoder [11], PMTA-AE-SC decoder [10], QOPCA-AE-SC decoder, and PPA-QOPCA-AE-SC decoder (θ=1/4) for the RM(8,3), RM(8,4), and RM(8,5) codes. (**a**) RM(8,3), n=256, k=93, and code rate = 0.36. (**b**) RM(8,4), n=256, k=163, and code rate = 0.64. (**c**) RM(8,5), n=256, k=219, and code rate = 0.86.

Figure 6 depicts the BLERs of various decoders for the three 256-length RM codes RM(8,3), RM(8,4), and RM(8,5). For the RM(8,3) and RM(8,4) codes, the AE-SC-256 decoder, which uses randomly sampled automorphisms from the whole general affine group of RM codes, demonstrated superior performance compared with the SCL-256 decoder and exhibited a higher rate of approaching the MLLB as the SNR increased. For the high-rate RM(8,5) code, both the AE-SC-256 decoder and the SCL-256 decoder achieved near-ML performances. It can be observed that the PMTA-AE-SC-256 decoder showed a nearly identical BLER to the AE-SC-256 decoder, which once again aligned with our analysis in Section 2.4. Notably, the proposed QOPCA-AE-SC-256 decoder exhibited negligible BLER degradation compared with the AE-SC-256 decoder for the RM(8,3) code. However, slight BLER losses of 0.13 dB and 0.17 dB were observed at a BLER of $2\times 10^{-5}$ for the RM(8,4) and RM(8,5) codes, respectively.

By comparing the BLER performance of the proposed QOPCA-AE-SC decoder for RM codes with various code rates, it can be observed that by utilizing a universal QOPC intensity threshold ω, the loss incurred by the QOPC-aided ET technique was more pronounced for the RM codes with higher code rates. This indicates that in the high-SNR regions where the high-rate code was effective, a large ω was required in the QOPCA-AE-SC decoder to ensure a high accuracy of the QOPC-based ET criterion to identify the best decoding path early. It is worth emphasizing that through a customized ω optimization for the high-rate RM codes, the BLER loss induced by the QOPC-aided ET criterion can be further mitigated.

### 4.2. Early Termination Gain

**Figure 7:**
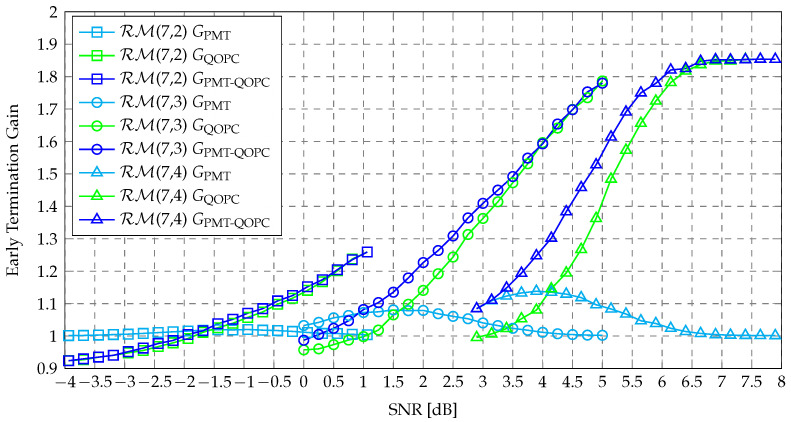
ETG comparison between the PMT-based [10], QOPC-based, and PMT-QOPC-based ET methods for the AE-SC-32 decoder of the RM(7,2), RM(7,3), and RM(7,4) codes.

**Figure 8:**
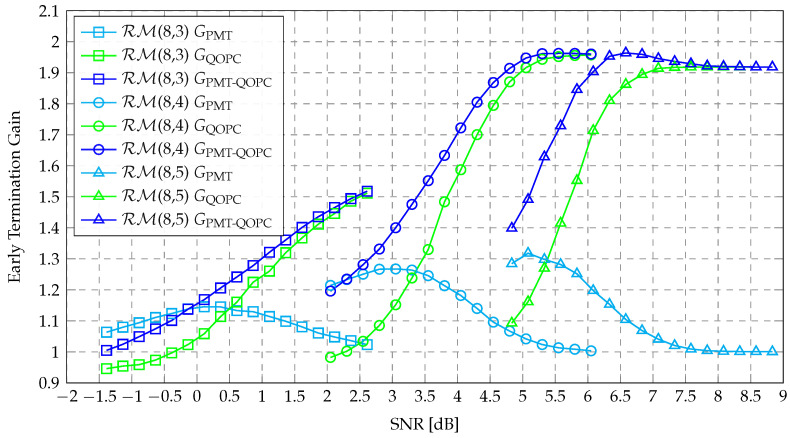
ETG comparison between the PMT-based [10], QOPC-based, and PMT-QOPC-based ET methods for the AE-SC-256 decoder of the RM(8,3), RM(8,4), and RM(8,5) codes.

The ETG metric, defined as the complexity ratio of the original AE-SC decoder to the AE-SC decoder using an ET technique, is initially introduced in [10] to quantify the effectiveness of ET methods. Let $G_{\mathrm{PMT}}$ and $G_{\mathrm{QOPC}}$ denote the ETG of the baseline PMTA-AE-SC decoder and the proposed QOPCA-AE-SC decoder, respectively. $G_{\mathrm{PMT}}$ is calculated using $G_{\mathrm{PMT}}=C^{\mathrm{OG}}/C^{\mathrm{PMT}}$, where $C^{\mathrm{PMT}}$ represents the average complexity of the PMTA-AE-SC decoder. Similarly, $G_{\mathrm{QOPC}}$ is calculated using $G_{\mathrm{QOPC}}=C^{\mathrm{OG}}/C^{\mathrm{QOPC}}$. Additionally, we use $C_{\mathrm{norm}}^{\mathrm{PMT}}$ and $C_{\mathrm{norm}}^{\mathrm{QOPC}}$ to represent the normalized complexity of the PMTA-AE-SC decoder and the QOPCA-AE-SC decoder, respectively. Specifically, $C_{\mathrm{norm}}^{\mathrm{PMT}}$ is defined as $C_{\mathrm{norm}}^{\mathrm{PMT}}=C^{\mathrm{PMT}}/C^{\mathrm{OG}}$, and $C_{\mathrm{norm}}^{\mathrm{QOPC}}$ is defined as $C_{\mathrm{norm}}^{\mathrm{QOPC}}=C^{\mathrm{QOPC}}/C^{\mathrm{OG}}$. It follows that $C_{\mathrm{norm}}^{\mathrm{PMT}}=1/G_{\mathrm{PMT}}$ and $C_{\mathrm{norm}}^{\mathrm{QOPC}}=1/G_{\mathrm{QOPC}}$. A higher ETG corresponds to a lower normalized complexity, both of which indicate a greater complexity reduction achieved by the ET technique. In Figure 7, we compare the ETG of the baseline PMTA-AE-SC-32 decoder [10] and the proposed QOPCA-AE-SC-32 decoder for the RM(7,2), RM(7,3), and RM(7,4) codes. Similarly, in Figure 8, we compare the ETG of the PMTA-AE-SC-256 decoder and the QOPCA-AE-SC-256 decoder for the RM(8,3), RM(8,4), and RM(8,5) codes.

As analyzed in Section 3.3.1, the ETG of the baseline PMTA-AE-SC decoder primarily occurs when only a small fraction of SC decoding paths generate PMs larger than the threshold *T*. This scenario typically corresponds to the low-to-medium SNR regions where the BLER is above $10^{-2}$. Table 2 summarizes the peak $G_{\mathrm{PMT}}$ achieved by the PMTA-AE-SC decoder for the tested RM codes and the corresponding SNR point at which it occurred. Using the SNR at which the peak $G_{\mathrm{PMT}}$ was achieved as the reference SNR, as the SNR lowered, it was highly probable that all SC decoding paths were terminated early due to a higher noise level, and as the SNR increased, more SC decoding paths tended to recover the codeword and generate PMs that did not exceed the threshold *T*. As a result, both Figure 7 and Figure 8 demonstrate that outside the SNR range where the peak $G_{\mathrm{PMT}}$ was obtained, the PMT-aided ET method gradually became less effective, and thus, exhibited a bell-shaped ETG curve.

Regarding the ETG of the proposed QOPC-aided ET method, it stems from the detection of the QOPC with a convergence intensity that exceeded the threshold ω, which occurred not only more frequently but also earlier during the AE-SC decoding at higher SNR regions. As illustrated in Figure 7 and Figure 8, as the SNR increased, the $G_{\mathrm{QOPC}}$ values of all the QOPCA-AE-SC decoders increased and consistently outperformed the PMTA-AE-SC decoder with notable improvements in high SNR regions. In addition, more significant ETG improvements were achieved for higher-rate RM codes with the QOPC-aided ET method, as the higher-rate RM codes were effective in higher-SNR regions.

Figure 7 and Figure 8 both demonstrate that the proposed QOPCA-AE-SC decoder tended to achieve a large ETG in high-SNR regions, while the baseline PMTA-AE-SC decoder was more effective under low-to-medium SNR ranges. To ensure a fair comparison between the two methods, we evaluated their ETGs at a fixed BLER of $10^{-3}$, which is a realistic working point for many communication systems [20]. Table 3 summarizes the ETG improvements achieved by our QOPC-aided ET method for the considered RM codes. The results indicate that the QOPCA-AE-SC decoder outperformed the baseline PMTA-AE-SC decoder across all the tested RM codes in terms of the ETG, with varying degrees of enhancement. Specifically, for the RM(7,3) and RM(7,4) codes, the proposed QOPCA-AE-SC-32 decoder reduced the normalized complexities to 74.6% and 56.5%, respectively, while the PMTA-AE-SC-32 decoder exhibited a minimal complexity reduction. For the RM(8,4) and RM(8,5) codes, the proposed QOPCA-AE-SC-256 decoder reduced the normalized complexities to 64.1% and 52.6%, respectively, whereas the PMTA-AE-SC-256 decoder exhibited high normalized complexities of 84.0% and 93.5%, respectively. However, for the low-rate RM(7,2) and RM(8,3) codes, the proposed QOPCA-AE-SC decoder provided only minor ETG improvements compared with the PMTA-AE-SC decoder.

Although the advantageous SNR regions of the proposed QOPC-aided ET method and the baseline PMT-aided ET method do not overlap, the two ET criteria can be employed simultaneously in the AE-SC decoding. In particular, the two ET methods are compatible in the following manner: If ω or more SC decoding paths survive the PMT-based ET criterion, the QOPC-based criterion can be invoked, resulting in a two-phase ET verification, which is referred to as the PMT-QOPC-based ET criterion. Let $G_{\mathrm{PMT}-\mathrm{QOPC}}$ denote the ETG achieved by the PMT-QOPC-based ET criterion. As depicted in Figure 7 and Figure 8, the PMT-QOPC-based criterion effectively combined the strengths of both approaches, where it achieved a higher ETG than the two independent techniques across a wide range of SNRs.

### 4.3. Strategies for Further ETG Enhancement

#### 4.3.1. Fine-Grained Parameter Optimization

As demonstrated in Figure 7 and Figure 8, the ETG growth rates of the QOPCA-AE-SC-32 decoder for the RM(7,4) code and the QOPCA-AE-SC-256 decoder for the RM(8,4) and RM(8,5) codes slowed down as the SNR increased to a certain high level. Eventually, their ETGs approached a maximum value near 2 and ceased to increase further. In fact, once the parameter λ was determined, $G_{\mathrm{QOPC}}$ became bounded, with its lower and upper bounds derived from Equations (13) and (14), respectively. Let $G_{\mathrm{QOPC}}^{\mathrm{low}}$ and $G_{\mathrm{QOPC}}^{\mathrm{up}}$ represent the lower and upper bounds of $G_{\mathrm{QOPC}}$. According to Equation (13), which defines the worst-case complexity $C_{\max}^{\mathrm{QOPC}}$ of the QOPCA-AE-SC decoder, $G_{\mathrm{QOPC}}^{\mathrm{low}}$ is expressed as

(17) $$G_{\mathrm{QOPC}}^{\mathrm{low}}=C^{\mathrm{OG}}/C_{\max}^{\mathrm{QOPC}}.$$

Similarly, based on Equation (14), which provides the best-case complexity $C_{\min}^{\mathrm{QOPC}}$, $G_{\mathrm{QOPC}}^{\mathrm{up}}$ is calculated using

(18) $$G_{\mathrm{QOPC}}^{\mathrm{up}}=C^{\mathrm{OG}}/C_{\min}^{\mathrm{QOPC}}.$$

$G_{\mathrm{QOPC}}^{\mathrm{up}}$ is of particular significance, as it governs the theoretical maximum achievable ETG of the proposed QOPC-based ET method. Table 4 provides a comprehensive summary of the key data used to derive $G_{\mathrm{QOPC}}^{\mathrm{up}}$ for the QOPCA-AE-SC decoders applied to the considered RM codes. As discussed in Section 3.3.1, $O_{n-1}^{\mathrm{SC}}$ denotes the total number of required *f* and *g* computations for a full SC decoding, while $O_{f_{n-k-1}}^{\mathrm{SC}}$ and $O_{f_{\lambda }}^{\mathrm{SC}}$ represent the number of *f* and *g* computations necessary for decoding the last frozen bit and the λ-th frozen bit, respectively. In this work, all these quantities were evaluated based on a non-parallelized SC decoding implementation.

Since the frozen set of the RM (*m*,*r*) code can be formulated by selecting bit indices with a Hamming weight less than m−r [11], $f_{n-k-1}$, which is the largest element in the frozen set of the RM (*m*,*r*) code, has a weight of m−r−1, with all the most significant m−r−1 bits set to 1 in its binary representation. From this perspective, the $f_{n-k-1}$ of the RM (*m*,*r*) code must be greater than the $f_{n-k-1}$ of the RM(*m*,r+1) code, i.e., the index of the last frozen bit is larger for lower-order (or lower-rate) RM codes with the same code length. Consequently, as illustrated in Table 4, the RM codes with lower orders exhibited $O_{f_{n-k-1}}^{\mathrm{SC}}$ values closer to $O_{n-1}^{\mathrm{SC}}$ since more nodes on the FFG were traversed during the decoding of the last frozen bit.

Additionally, leveraging the well-established Plotkin construction of RM codes, the RM (*m*,*r*) code can be recursively partitioned into two subcodes: RM(m−1,r−1) and RM(m−1,*r*) [22]. Since the RM(m−1,r−1) subcode can be characterized by the SRUS of the RM (*m*,*r*) code, the selected λ from the proposed SRUS-based rule corresponds to the bit index of the last frozen bit of the RM(m−1,r−1) subcode. Consequently, λ is inherently larger for lower-order RM codes, as their subcodes also exhibit lower orders.

Table 4 demonstrates that for the considered RM codes, $O_{f_{n-k-1}}^{\mathrm{SC}}$ and $O_{n-1}^{\mathrm{SC}}$ were of the same order of magnitude. Additionally, from the subcode perspective previously discussed, $O_{f_{\lambda }}^{\mathrm{SC}}$ was roughly half of $O_{n-1}^{\mathrm{SC}}$. Although different RM codes exhibited variations in $O_{f_{n-k-1}}^{\mathrm{SC}}$ and $O_{f_{\lambda }}^{\mathrm{SC}}$, the ratio of $O_{f_{n-k-1}}^{\mathrm{SC}}$ to $O_{f_{\lambda }}^{\mathrm{SC}}$ remained stable across all the tested RM codes, where it consistently approached a value near 2. By comparing Equations (14) and (15), we observed that when a large automorphism ensemble size *M* was used in the AE-SC decoding, such as M=32 and M=256 in our experiments, $G_{\mathrm{QOPC}}^{\mathrm{up}}$ was primarily governed by the ratio of $O_{f_{n-k-1}}^{\mathrm{SC}}$ to $O_{f_{\lambda }}^{\mathrm{SC}}$. This explains why $G_{\mathrm{QOPC}}$ for decoding the RM(7,4), RM(8,4), and RM(8,5) codes attained a maximum value near 2. Notably, as illustrated in Figure 8, $G_{\mathrm{PMT}-\mathrm{QOPC}}$ surpassed $G_{\mathrm{QOPC}}^{\mathrm{up}}$ for the RM(8,5) code due to the additional ETG provided by the PMT-based ET method. However, as the SNR increased, $G_{\mathrm{PMT}-\mathrm{QOPC}}$ decreased to the level of $G_{\mathrm{QOPC}}^{\mathrm{up}}$, as the PMT-based ET method became less effective with a further reduction in the noise level.

The λ and ω selection strategies introduced in Section 3.3 balance the trade-off between the ETG and error-correction performances while maintaining the generalizability of the method. According to the proposed SRUS rule, the parameter λ, along with $O_{f_{\lambda }}^{\mathrm{SC}}$, is uniquely determined for each RM code. Thus, λ solely dominates $G_{\mathrm{QOPC}}^{\mathrm{up}}$ under fully parallel AE-SC decoding. To further increase the maximum achievable ETG, a feasible approach is to perform fine-grained joint optimization of λ and ω for the QOPCA-AE-SC decoder, enabling the use of a smaller λ and thereby enhancing $G_{\mathrm{QOPC}}^{\mathrm{up}}$.

#### 4.3.2. Partially Parallel Adaptive Decoding

An alternative method to enhance $G_{\mathrm{QOPC}}^{\mathrm{up}}$ is to apply the proposed QOPC-based ET technique within a partially parallel AE-SC decoding framework. The partially parallel AE-SC decoding maintains the same space complexity as the fully parallel AE-SC decoding but divides the *M* SC decoding paths into multiple sequentially launched groups. The proposed QOPC-based ET verification is performed across all launched path groups. Once the QOPC-based ET condition is met during the execution of any path group, the output path is determined, and the remaining path groups are skipped. Since the QOPC-based ET criterion may be satisfied within the first path group, $G_{\mathrm{QOPC}}^{\mathrm{up}}$ can be significantly elevated.

Based on the above observation, we introduce a partially parallel adaptive QOPCA-AE-SC (PPA-QOPCA-AE-SC) decoder, which employs a simple path grouping strategy by dividing the *M* SC decoding paths into two groups. The PPA-QOPCA-AE-SC decoder uses a parameter θ for parallelism control, where 0<θ≤1 and ⌊M×θ⌋≥ω. The first ⌊M×θ⌋ SC decoding paths form the first group, while the remaining M−⌊M×θ⌋ paths constitute the second group. Specifically, the PPA-QOPCA-AE-SC decoder operates as follows: The first group is executed first. If the QOPC-based ET criterion is satisfied in the first group, the output path is determined, and the second group is skipped. If the ET condition is not met in the first group, the second group is launched, which is equivalent to performing the QOPC-based ET verification in the fully parallel QOPCA-AE-SC decoding by reusing the stored PPM data from the first group.

It is worth noting that the PPA-QOPCA-AE-SC decoder does not involve additional parameter optimization. Particularly, it shares the QOPC intensity threshold ω with its non-adaptive counterpart, the fully parallel QOPCA-AE-SC decoder. This design is motivated by the fact that the first executed path group only observes a subset of *M* SC decoding paths. To ensure sufficient confidence in determining the output path when the QOPC-based ET criterion is satisfied within the first path group, a relatively high QOPC intensity threshold should be set for the ET verification. Although the first path group has an ensemble size smaller than the full parallelism *M*, it retains the same QOPC intensity threshold ω originally optimized for the fully parallel QOPCA-AE-SC decoder. This approach increases the likelihood of consistency between the output paths determined from the locally triggered and globally triggered ET verifications, thereby preserving the error-correction performance.

For the AE-SC decoding, full parallelism is often unnecessary in the high-SNR region, where partial parallelism typically provides sufficient diversity gain for error correction. By employing the progressive path-launching mechanism, the proposed PPA-QOPCA-AE-SC decoder can adapt to noise levels and dynamically adjust the decoding complexity. Let $C^{\mathrm{PPA}-\mathrm{QOPC}}$ and $G_{\mathrm{PPA}-\mathrm{QOPC}}$ denote the average complexity and the ETG of the PPA-QOPCA-AE-SC decoder, respectively. According to the definition of ETG, we have $G_{\mathrm{PPA}-\mathrm{QOPC}}=C^{\mathrm{OG}}/C^{\mathrm{PPA}-\mathrm{QOPC}}$. Additionally, we use $C_{\mathrm{norm}}^{\mathrm{PPA}-\mathrm{QOPC}}$ to represent the normalized complexity of the PPA-QOPCA-AE-SC decoder, which is calculated as $C_{\mathrm{norm}}^{\mathrm{PPA}-\mathrm{QOPC}}=1/G_{\mathrm{PPA}-\mathrm{QOPC}}$. The minimum value of $C^{\mathrm{PPA}-\mathrm{QOPC}}$, denoted as $C_{\min}^{\mathrm{PPA}-\mathrm{QOPC}}$, occurs when the QOPC-based ET criterion is consistently satisfied at the λ-th frozen bit within the first path group, and is given by

(19) $$C_{\min}^{\mathrm{PPA}-\mathrm{QOPC}}=O_{n-1}^{\mathrm{SC}}+O_{f_{\lambda }}^{\mathrm{SC}}\cdot \left(\left\lfloor M\times \theta \right\rfloor -1\right)+\left\lfloor M\times \theta \right\rfloor +1.$$

Furthermore, the upper bound of $G_{\mathrm{PPA}-\mathrm{QOPC}}$, denoted as $G_{\mathrm{PPA}-\mathrm{QOPC}}^{\mathrm{up}}$, is calculated using

(20) $$G_{\mathrm{PPA}-\mathrm{QOPC}}^{\mathrm{up}}=C^{\mathrm{OG}}/C_{\min}^{\mathrm{PPA}-\mathrm{QOPC}}.$$

Since $C_{\min}^{\mathrm{PPA}-\mathrm{QOPC}}$ is approximately $C_{\min}^{\mathrm{QOPC}}\times \theta$ for large *M* in AE-SC decoding, $G_{\mathrm{PPA}-\mathrm{QOPC}}^{\mathrm{up}}$ is roughly $G_{\mathrm{QOPC}}^{\mathrm{up}}/\theta$. Consequently, $G_{\mathrm{PPA}-\mathrm{QOPC}}^{\mathrm{up}}$ significantly outperforms $G_{\mathrm{QOPC}}^{\mathrm{up}}$ when a small θ is used in the PPA-QOPCA-AE-SC decoder.

**Figure 9:**
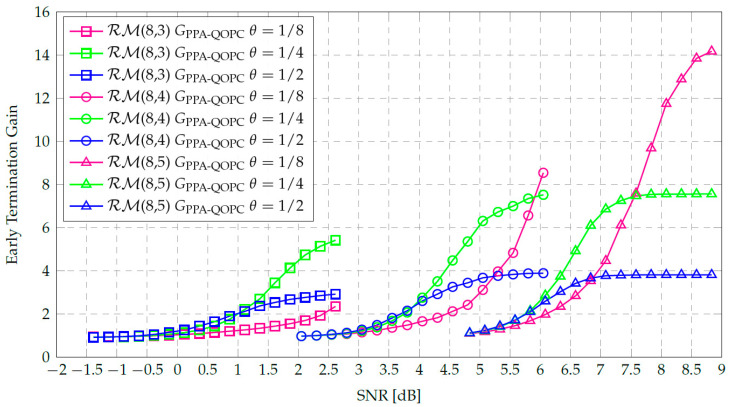
ETGs of the PPA-QOPCA-AE-SC-256 decoder with various θ values for decoding the RM(8,3), RM(8,4), and RM(8,5) codes.

Figure 9 illustrates the ETGs of the PPA-QOPCA-AE-SC-256 decoder for the RM(8,3), RM(8,4), and RM(8,5) codes, with θ values of 1/8, 1/4, and 1/2. It can be observed that $G_{\mathrm{PPA}-\mathrm{QOPC}}$ exceeded $G_{\mathrm{QOPC}}^{\mathrm{up}}$ for all three 256-length RM codes, which validated the effectiveness of the adaptive parallelism scheduling in achieving an ETG beyond $G_{\mathrm{QOPC}}^{\mathrm{up}}$. Since a smaller θ makes the QOPC-based ET condition of the first path group more stringent, and thus, harder to satisfy, the PPA-QOPCA-AE-SC-256 decoder using a small θ of 1/8 only showed significant ETG growth in the high-SNR region. In contrast, a larger θ made the QOPC-based ET condition more likely to be met within the first path group, so the ETG was more noticeable in the low-to-medium SNR regions, as seen with θ values of 1/4 and 1/2. However, $G_{\mathrm{PPA}-\mathrm{QOPC}}^{\mathrm{up}}$ was also smaller with a larger θ.

Overall, the PPA-QOPCA-AE-SC-256 decoder with θ=1/4 achieved the best ETG performance across a wide range of SNR for the three 256-length RM codes. Moreover, the simulation results in Figure 6 show that the PPA-QOPCA-AE-SC-256 decoder that used θ=1/4 achieved a nearly identical BLER performance to the QOPCA-AE-SC-256 decoder. This confirmed that utilizing the same parameters for QOPC-based ET verification in the first path group as in fully parallel QOPCA-AE-SC decoding was indeed effective. Table 5 summarizes the ETG improvements achieved by the PPA-QOPCA-AE-SC decoder with θ=1/4 at a BLER near $10^{-5}$. Specifically, the fully parallel QOPCA-AE-SC-256 decoder reduced the normalized complexities to 74.6%, 54.6%, and 52.1% for the RM(8,3), RM(8,4), and RM(8,5) codes, respectively. The PPA-QOPCA-AE-SC-256 decoder further reduced the normalized complexities to significantly lower values of 32.5%, 18.7%, and 13.3%, respectively. As $G_{\mathrm{PPA}-\mathrm{QOPC}}$ approached $G_{\mathrm{PPA}-\mathrm{QOPC}}^{\mathrm{up}}$ in the high-SNR region, more pronounced ETG improvements were achieved by the PPA-QOPCA-AE-SC decoder for the higher-rate RM codes.

The underlying logic of the PPA-QOPCA-AE-SC decoder closely resembles that of the adaptive SCL decoder [23]. The first launched path group in the PPA-QOPCA-AE-SC decoder essentially forms a QOPCA-AE-SC decoder with an automorphism ensemble size of ⌊M×θ⌋, offering lower decoding complexity but weaker error-correction capability. The role of the QOPC-based ET verification is analogous to the cyclic redundancy check (CRC) in the adaptive SCL algorithm [24]. When the QOPC-based ET criterion is satisfied within the first path group, it provides high confidence that the correct decoding path has been found. If the low-complexity partially parallel decoding fails to trigger the QOPC-based ET criterion, a more powerful QOPCA-AE-SC decoder with full parallelism is activated for decoding.

Notably, the worst-case complexity of the PPA-QOPCA-AE-SC decoder is nearly identical to that of the non-adaptive QOPCA-AE-SC decoder, with only a minor complexity overhead introduced by the QOPC-based ET verification during the first path group decoding. In other words, the PPA-QOPCA-AE-SC decoder progressively increases its complexity to enhance the decoding capability while maintaining a stable worst-case complexity regardless of the number of path groups, as each path group is launched only once. However, due to the sequential manner of the path group execution, the PPA-QOPCA-AE-SC decoder exhibits dynamic latency, with the worst-case latency being twice that of the fully parallel QOPCA-AE-SC decoding.

### 4.4. Extensibility and Practical Relevance

The origin of the QOPC phenomenon stems from the automorphism ensemble decoding framework and the SC constituent decoders adopted. Although we focus on RM codes to validate the effectiveness of the proposed QOPCA-AE-SC decoder, the QOPC-aided ET method is applicable to the AE-SC decoding of any polar-like codes that exhibit rich SC-variant automorphisms [13,25,26]. This offers a universal solution for implementing complexity-saving strategies across diverse coding schemes.

For simplicity, this work adopts the non-parallelized version of SC decoding to quantify and evaluate the complexity of AE-SC decoding. With minor modifications to the PM formulation, the proposed QOPCA-AE-SC decoder is inherently compatible with advanced node-based fast decoding techniques [27]. This highlights the potential of our approach to be applied in AE-SC decoding with further reduced latency and complexity, making it suitable for more stringent latency-critical or power-constrained applications.

Since the proposed QOPC-aided ET method relies on straightforward threshold comparisons, it achieves ETG improvements over the baseline PMT-aided ET method without significantly increasing the computational costs. Apart from the ETG improvements, the QOPC-aided ET method exhibits lower sensitivity to quantization noise in channel LLRs, as it eliminates the reliance on prior SNR information and leverages the relative characteristics of the PPMs rather than their absolute values in SC decoding. Thus, the QOPC-aided ET method offers greater stability than the PMT-aided ET method in quantized hardware-based AE-SC decoders.

While we focused on analysis and simulations in this study, future work aims to further validate the practical applicability of our method from the following two aspects:

1. Explore the QOPCA-AE-SC decoder implementation on application-specific integrated circuit (ASIC) or field-programmable gate array (FPGA) platforms to analyze its power consumption and scalability;
2. Evaluate the QOPCA-AE-SC decoding performance in real-time scenarios on a software-defined radio (SDR) platform and test it over diverse channel models, such as Rayleigh fading and Rician fading, to assess its adaptability over different real-time communication channels.

## 5. Conclusions

In this paper, we introduce a novel quasi-optimal path convergence (QOPC)-aided early termination (ET) technique for the AE-SC decoding of RM codes, which reduces the overall complexity without compromising the error-correction performance. The proposed QOPC-aided ET method monitors the partial path metrics (PPMs) of SC decoding paths to detect the sufficiently intense QOPC phenomenon, enabling reliable early identification of the best decoding path at runtime. When the QOPC-based ET criterion is satisfied during AE-SC decoding, only the identified path is allowed to proceed for a complete codeword estimate, while the remaining SC decoding paths are deactivated early. Numerical results demonstrated that for multiple RM codes with varying lengths and rates, the proposed QOPC-aided ET method incurred minimal BLER losses across a wide range of SNRs under fully parallel AE-SC decoding while achieving greater complexity reduction at a lower BLER. Specifically, for medium-to-high-rate RM codes, the QOPC-aided ET method achieved a complexity reduction that ranged from 35.9% to 47.4% at a BLER of $10^{-3}$, which consistently outperformed the state-of-the-art path metric threshold (PMT)-aided ET method. Additionally, at a lower BLER that approached $10^{-5}$, the proposed QOPC-aided ET method achieved a more substantial complexity reduction that ranged from 81.3% to 86.7% when applied to a partially parallel framework of AE-SC decoding. The proposed QOPC-based ET technique is compatible with existing complexity- and latency-reduction techniques and can be utilized for power reduction in hardware AE-SC decoders. Future work plans to further validate the practical applicability of our method through hardware implementations and real-time testing over diverse communication channels.

## Figures and Tables

**Table 1:** Code parameters of the considered RM codes and the algorithmic configurations for the proposed QOPCA-AE-SC decoder and the baseline PMTA-AE-SC decoder.

Code	*n*	*k*	Rate	*M*	λ	ω	*T* (SNR [dB])
RM(7,2)	128	29	0.23	32	56	16	−41.4(−0.8)
RM(7,3)	128	64	0.50	32	41	16	−29.9(3.0)
RM(7,4)	128	99	0.77	32	21	16	−14.7(6.2)
RM(8,3)	256	93	0.36	256	98	32	−65.6(0.7)
RM(8,4)	256	163	0.64	256	63	32	−42.3(3.9)
RM(8,5)	256	219	0.86	256	28	32	−19.4(6.7)

**Table 2:** The peak ETG of the baseline PMTA-AE-SC-*M* decoder and the corresponding SNR point at which it occurred.

Code	*n*	*k*	Rate	*M*	SNR [dB]	$G_{\mathrm{PMT}}$
RM(7,2)	128	29	0.23	32	−1.44	1.02
RM(7,3)	128	64	0.50	32	1.50	1.08
RM(7,4)	128	99	0.77	32	3.89	1.14
RM(8,3)	256	93	0.36	256	0.11	1.15
RM(8,4)	256	163	0.64	256	3.05	1.27
RM(8,5)	256	219	0.86	256	5.08	1.32

**Table 3:** ETG comparison between the proposed QOPCA-AE-SC decoder and the baseline PMTA-AE-SC decoder at the BLER of $10^{-3}$.

Code	*n*	*k*	Rate	*M*	SNR [dB]	$G_{\mathrm{PMT}}$ ($C_{\mathrm{norm}}^{\mathrm{PMT}}$)	$G_{\mathrm{QOPC}}$ ($C_{\mathrm{norm}}^{\mathrm{QOPC}}$)
RM(7,2)	128	29	0.23	32	−0.75	1.02 (98.0%)	1.07 (93.5%)
RM(7,3)	128	64	0.50	32	2.9	1.04 (96.2%)	1.34 (74.6%)
RM(7,4)	128	99	0.77	32	6.1	1.02 (98.0%)	1.77 (56.5%)
RM(8,3)	256	93	0.36	256	0.7	1.13 (88.5%)	1.19 (84.0%)
RM(8,4)	256	163	0.64	256	3.9	1.19 (84.0%)	1.56 (64.1%)
RM(8,5)	256	219	0.86	256	6.8	1.07 (93.5%)	1.90 (52.6%)

**Table 4:** $G_{\mathrm{QOPC}}^{\mathrm{up}}$ values of the evaluated QOPCA-AE-SC decoders for the considered RM codes.

Code	*n*	*k*	Rate	*M*	λ	$O_{n-1}^{\mathrm{SC}}$	$O_{f_{n-k-1}}^{\mathrm{SC}}$	$O_{f_{\lambda }}^{\mathrm{SC}}$	$G_{\mathrm{QOPC}}^{\mathrm{up}}$
RM(7,2)	128	29	0.23	32	56	896	879	443	1.92
RM(7,3)	128	64	0.50	32	41	896	847	431	1.90
RM(7,4)	128	99	0.77	32	21	896	767	399	1.86
RM(8,3)	256	93	0.36	256	98	2048	1999	1007	1.98
RM(8,4)	256	163	0.64	256	63	2048	1919	975	1.96
RM(8,5)	256	219	0.86	256	28	2048	1727	895	1.92

**Table 5:** ETG comparison between the fully parallel QOPCA-AE-SC-256 decoder and the PPA-QOPCA-AE-SC-256 decoder (θ=1/4) for the tested 256-length RM codes at a BLER of near $10^{-5}$.

Code	*n*	*k*	Rate	SNR [dB]	$G_{\mathrm{QOPC}}$ ($C_{\mathrm{norm}}^{\mathrm{QOPC}}$)	$G_{\mathrm{PPA}-\mathrm{QOPC}}$ ($C_{\mathrm{norm}}^{\mathrm{PPA}-\mathrm{QOPC}}$)
RM(8,3)	256	93	0.36	1.5	1.34 (74.6%)	3.08 (32.5%)
RM(8,4)	256	163	0.64	4.7	1.83 (54.6%)	5.36 (18.7%)
RM(8,5)	256	219	0.86	7.7	1.92 (52.1%)	7.51 (13.3%)

## Data Availability

The original contributions found in this study are included in this article. Further inquiries can be directed to the corresponding author.
